# An Oligodeoxynucleotide with Promising Modulation Activity for the Proliferation and Activation of Osteoblast

**DOI:** 10.3390/ijms12042543

**Published:** 2011-04-15

**Authors:** Zhiyuan Feng, Yuqin Shen, Liying Wang, Lin Cheng, Jing Wang, Quanshun Li, Wei Shi, Xinhua Sun

**Affiliations:** 1 Department of Orthodontics, School of Stomatology, Jilin University, 1500 Qinghua Road, Changchun 130021, China; E-Mails: fzy00@sina.com (Z.F.); 33817805@qq.com (J.W.); 2 Department of Periodontal, School of Stomatology, Jilin University, 1500 Qinghua Road, Changchun 130021, China; E-Mail: shenyuqin326@sina.com; 3 Department of Molecular Biology, College of Basic Medicine, Jilin University, Changchun 130021, China; E-Mail: wlying@jlu.edu.cn; 4 Department of Oral Cavity, The Second Affiliated Hospital of Shanxi Medical University, 382 Wuyi Road, Taiyuan 030001, China; E-Mail: chenglin6002@sina.com; 5 Key Laboratory for Molecular Enzymology and Engineering of Ministry of Education, Jilin University, 2699 Qianjin Road, Changchun 130021, China; E-Mail: quanshun@jlu.edu.cn

**Keywords:** oligodeoxynucleotide, osteoblast, proliferation, activation, bone remodeling

## Abstract

The paper explored the regulatory role of oligodeoxynucleotides (ODNs) with specific sequences in the proliferation and activation of osteoblast, using human osteoblast-like cell line MG 63 as the model. Through the administration of ODNs to MG 63 cells at a concentration of 1.0 μg/mL, ODN MT01 with positive effects on proliferation and activation of osteoblast was selected from 11 different ODNs by methyl thiazolyl tetrazolium (MTT) assay and alkaline phosphatase (ALP) activity measurement. To get a deeper insight into the molecular mechanism, effects of ODN MT01 treatment on the expression level of Sp7, runx-2, collagen-I, osteoprotegerin (OPG) and RANK ligand (RANKL) were determined using quantitative real time PCR and Western blotting. Remarkably, the mRNA and protein expression levels of Sp7, runx-2, collagen-I and OPG were improved after ODN MT01 treatment. Meanwhile, the protein expression level of RANKL was dramatically decreased. These results suggested that ODN MT01 had a significant impact in facilitating osteogenic proliferation and activation, and provided a direct evidence for the notion that single strand ODN could regulate the balance of bone formation and resorption, and thus was of great potential in the rebuilding of alveolar bone.

## 1. Introduction

Orthodontic tooth movement is closely related to the remodeling of alveolar bone, and alveolar bone loss always occurs during orthodontic tooth movement, especially in patients with periodontal disease [1,2]. In addition, the loose and even loss of teeth caused by periodontal disease affects quality of life, even to the extent of forming edentulous jaw. For the edentulous patients with severe alveolar bone absorption, good retention and aesthetics could not be achieved only by dentures. Therefore, the regeneration of alveolar bone is of great significance in oral medicine research.

In physiological conditions, the bone remodeling process is controlled by a complex network of endocrine hormones and local cytokines [3,4]. Two types of cells are involved in bone remodeling: osteoclasts originating from hematopoietic cells are responsible for bone resorption, and osteoblasts originating from mesenchymal cells are responsible for the formation of new bone [5]. Generally, the regeneration of alveolar bone processes as follows: growth factor-inducing bone marrow mesenchymal stem cells or periodontal ligament stem cells differentiate into osteoblast, which then synthesizes the mineralized bone matrix, and ultimately induces the formation of bone in a complex biological route [6]. Over the past few years, research on alveolar bone regeneration has been greatly developed from the general tissue and cell levels to protein and gene levels [7,8]. With the development of alveolar surgery, molecular biology and tissue engineering, more and more new technology has been used for the repair and regeneration of alveolar bone defects, such as distraction osteogenesis [9,10], guided bone regeneration membrane [11], and application of growth factors [12,13]. Since osteoblast is directly associated with the synthesis of mineralized bone matrix, the proliferation and activation of osteoblast using growth factors and other agents will be an attractive issue in the alveolar bone repair and regeneration.

Oligodeoxynucleotide (ODN) is one kind of the unmethylated nucleotide core motif sequences, which are unusual in mammalian genomes but prevalent in prokaryotes. It can also be obtained from the DNA degradation products, and even synthesized and modified in an artificial way. It is usually absorbed by cells in an endocytic manner, and then activates the pathway involving Toll-like receptor-9 (TLR9) [14]. Recent studies have found that specific ODN could regulate the formation and activation of osteoclasts [15–17]. Amcheslavsky *et al*. reported the effects of ODN on two types of mice osteoclast precursor cells (rich in TLR9 and TLR9 deficient, respectively), and concluded that ODN played a key role in regulating the formation and differentiation of osteoclast in a TLR9 dependent manner [16]. Chang *et al.* proved that the recognition of ODN CpG-KSK13 via TLR9 could inhibit the osteoclastogenesis by down-regulating the expression of TREM-2 [17]. In 2003, Penolazzi *et al.* found that a specific sequence of ODN could counteract nuclear factor-κB (NF-κB) to promote the osteoclast apoptosis and inhibit the early differentiation of osteoclast [15]. The receptor activators of NF-κB (RANK), RANK ligand (RANKL) and osteoprotegerin (OPG), constitute the essential regulatory components in the paracrine signaling necessary for osteoclast differentiation, activation and apoptosis, which has been considered as a connection pathway between osteoblast and osteoclastogenesis [18]. Previous research showed that CpG ODNs could modulate the osteoclastogenesis in bone marrow cell/osteoblast co-cultures [19]. As the osteoblast could express the receptor TLR9, upon TLR9 ligation, the RANKL expression level was improved in osteoblast, and could be inhibited by chloroquine, which proved that CpG ODNs induced the osteoclastogenic activity in osteoblast in a RANKL-dependent pattern [19]. However, the effects of ODNs on the activation and proliferation of osteoblast have not been clearly elucidated.

In the present research, we designed and synthesized 11 ODNs of different sequences, and those that could promote the proliferation of osteoblast-like cell line MG 63 were screened by methyl thiazolyl tetrazolium (MTT) assay. Then the ODNs with the ability to promote osteoblastic proliferation and activation were selected by the detection of alkaline phosphatase (ALP) expression level. Using real time PCR and Western blotting analysis, effects of ODNs on the expression level of Sp7, runx-2, collagen-I, OPG and RANKL were investigated to get a deeper insight into the molecular mechanism.

## 2. Results and Discussion

### 2.1. MTT Assay

**Figure 1.**
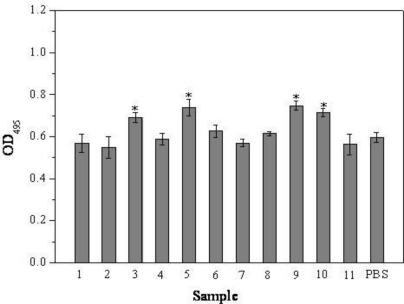
Effects of 11 different ODNs on the cell viability and proliferation of MG 63 osteoblast-like cells after 72 h treatment. *Statistically significant difference (*P* < 0.05) between experimental and control groups.

In the present research, we first investigated the effects of 11 different ODNs on the cell viability and proliferation of MG63 osteoblast-like cells using MTT assay (Figure 1). It was shown that the OD_495_ values of cells co-cultured with ODN BW001, ODN FC002, ODN MT01, ODN YW001 were higher than the control group. After 72 h, compared with the control, the values increased by 15.9%, 23.7%, 25.4% and 19.8%, respectively (*P* < 0.05). Similar tendency was observed for other treatment time (data not shown). The results indicated that the proliferation efficiency were highly associated with the sequence, and ODN BW001, ODN FC002, ODN MT01 and ODN YW001 could efficiently promote the early proliferation of MG 63 cells in this research.

Previous reports have shown that ODNs could be absorbed into cells through the receptor-mediated pathway without constructing a vector, and applied for evaluating the curative effect on treatment mastitis, allergic rhinitis and conjunctivitis in pre-clinical studies. In addition, specific ODNs could regulate the osteoclast differentiation through different signaling pathways [20,21]. Herein, we modified all the ODNs via phosphorothioic acid to enhance their cellular uptake capability and stability. The above results showed that ODNs of specific sequences could influence the proliferation of osteoblast and further regulate the osteoblast-osteoclast balance through different signaling pathways, which provided a potential way for regulating bone rebuilding using ODNs.

### 2.2. Effect of ODNs on ALP Activity

**Figure 2.**
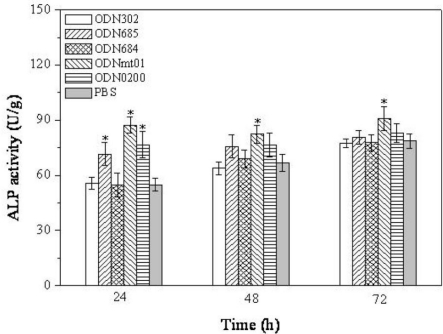
Effect of ODNs treatment on ALP activity of MG 63 cells, for different time intervals. *Statistically significant difference (*P* < 0.05) between experimental and control groups at the given time point.

To assess the effect of ODNs on osteoblastic activation, MG 63 cells were treated with five ODNs (ODN BW001, ODN FC002, ODN BW006, ODN MT01 and ODN YW001) at a concentration of 1.0 μg/mL, which have been screened from the MTT assay, and then the activities of osteoblast-related marker ALP were assessed. As shown in Figure 2, the ALP activities were associated with the sequence of ODNs and treatment time. At 24 h, the experimental groups of ODN FC002, ODN MT01, ODN YW001 significantly induced ALP activity in MG 63 (*P* < 0.05). However, after 48 h and 72 h treatment, there were no statistically significant differences between the experimental groups (ODN FC002 and ODN YW001) and control group. However, the ALP activities were remarkably improved compared to the control group after ODN MT01 treatment, with statistically significant differences at 24 h, 48 h and 72 h (*P* < 0.05).

To date, most research associated with ODN focused on the CG-rich motif ODN (CpG ODN). Recently, Zou *et al.* found that CpG ODN could regulate the osteoclast formation and differentiation in TLR9 dependent individual through TLR9-mediated molecular pathway [19]. CpG ODN could inhibit the osteoclast differentiation by upregulating the expression of IL-12 to inhibit RANKL, which suggested its potential in preventing the possibility of pathological bone resorption [22]. However, there are no previous reports that ODNs with no CpG motif had effects on the activation and proliferation of osteoblasts. In this paper, using MTT and ALP assay, we first obtained the no CpG ODNs with promising activity for promoting the proliferation and activation of MG 63 cells, and thus provided a novel way for the bone rebuilding research.

### 2.3. Effect of ODN MT01 on the Morphology of MG 63 Cells

**Figure 3.**
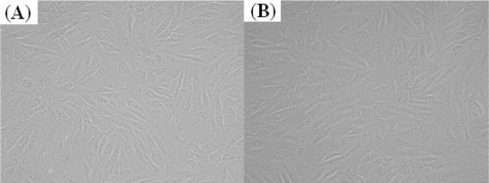
(**A**) MG 63 cells cultured in DMEM for 72 h; (**B**) MG 63 cells cultured in ODN MT01 (1.0 μg/mL)-DMEM for 72 h.

After treatment with 1.0 μg/mL ODN MT01 for 72 h, we used an inverted microscope to evaluate the effect of ODN MT01 on the cellular morphology of MG 63. As shown in Figure 3, good growth state was observed in both the ODN MT01-treated group and control group, which indicated that there were no adverse effects with ODN MT01 treatment.

### 2.4. Effect of ODN MT01 on Gene Expression of MG 63 Cells

**Figure 4.**
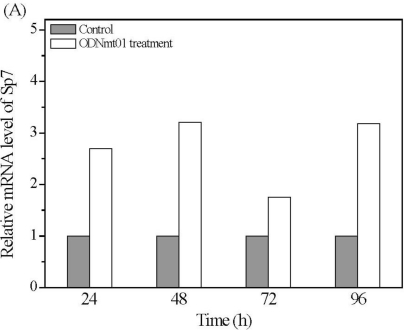

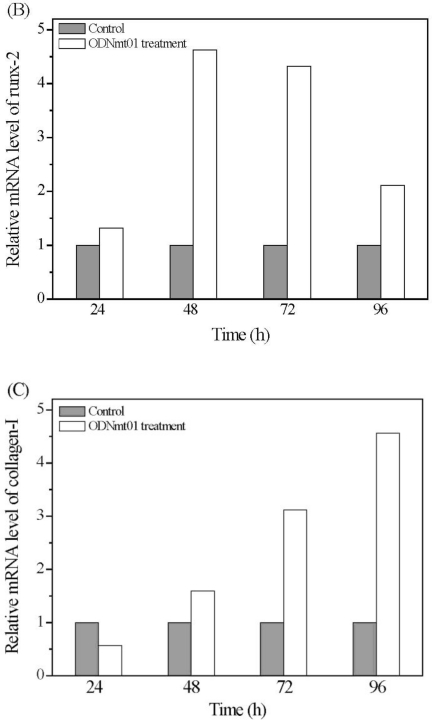
Real time PCR analysis of MG 63 cells of experimental group (ODN MT01 treatment) and control group (PBS treatment) at predetermined time. (**A**) Relative amounts of mRNA for Sp7 were quantified and GAPDH was used as internal control; (**B**) Relative amounts of mRNA for runx-2 were quantified and GAPDH was used as internal control; (**C**) Relative amounts of mRNA for collagen-I were quantified and GAPDH was used as internal control. *Statistically significant difference (*P* < 0.05) between experimental and control groups (*n* = 3).

The above results have confirmed that ODN MT01 could efficiently promote the proliferation and activation of osteoblast. Afterwards, we detected the effects of ODN MT01 treatment on the mRNA level of bone-related factors Sp7, runx-2, and collagen-I, using quantitative real time PCR. As shown in Figure 4, compared with control group, mRNA levels of Sp7, runx-2 and collagen-I were increased in MG 63 cells after ODN MT01 treatment. For Sp7, significantly higher mRNA levels were observed at 24 h, 48 h and 96 h after ODN MT01 treatment (Figure 4A); for runx-2 mRNA level, it exhibited an increasing tendency before 48 h and then slightly decreased (Figure 4B). Nevertheless, the collagen-I mRNA level was upregulated in the experimental group with the increase of culture time, and the values were much higher than those of control group after 72 h and 96 h treatment, which provided a direct evidence for the up-regulation of osteoblastic activity (Figure 4C).

The transcription factor runx-2 has a well-defined role in mediating the final stages of osteoblastic maturation, and is necessary for the normal osteogenesis and usually upregulated during osteoblastic activity. Runx-2 deficiency or mutation could cause severe bone abnormalities in mouse and human [23,24]. In runx-2 null mice, no endochondral or membranous bone was formed, due to an arrest during the early steps of osteoblast differentiation. Here, the mRNA level of runx-2 was greatly influenced by ODN MT01, and higher expression levels at 48 h and 72 h proved that ODN MT01 could promote the early osteoblastic activation of MG 63 cells. Sp7, another transcription factor, was considered as the downstream of runx-2, and could served as an inhibitor of chondrogenesis and chondrocyte maturation, while it promoted the osteoblast maturation [25,26]. Meanwhile, Sp7 expression was up-regulated by BMP-2 in a runx-2-independent pattern [27]. The above results suggested Sp7 might function as a master regulator capable of converting non-osseous mesenchymal cells (e.g., fibroblasts) into cells committed to the osteogenic lineage (*i.e.*, osteoblasts). In addition, Kim *et al.* demonstrated that the forced expression of Sp7 was not sufficient to convert non-osseous cells into osteoblastic cells, and promote full expression of the mature bone phenotype [28]. In our study, the results showed that ODN MT01 could significantly increase the mRNA level of Sp7, runx-2 and collagen-I at an early stage, which suggested that the proliferation and activation of MG 63 cells after ODN MT01 treatment were probably attributed to the abnormal expression of these osteoblast-related factors.

### 2.5. Western Blotting Analysis

**Figure 5.**
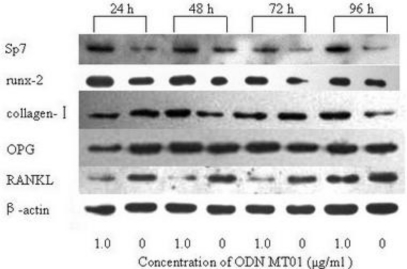
Western blotting analysis of expression level of Sp7, runx-2, collagen-I, OPG and RANKL in ODN MT01 treatment group and control group at predetermined time.

To get a further insight, MG 63 was treated with ODN MT01 (1.0 μg/mL) for different time, the protein expression levels of Sp7, runx-2, collagen-I, OPG and RANKL were measured using Western blotting technique. As shown in Figure 5, compared with the control group, the protein expression levels of Sp7, runx-2 and collagen-I were obviously increased to a certain degree. Significantly higher expression levels of Sp7 were observed at 24 h and 48 h in the experimental group, and then decreased slightly at 72 h and increased at 96 h, which was consistent with real time PCR analysis. The protein expression levels of runx-2 were slightly increased from 24 to 96 h, and the collagen-I expression levels of the experimental group were significantly higher at 48 h and 96 h. Meanwhile, the OPG expression levels of experimental group were higher at 48 h and 72 h. Nevertheless, RANKL expression level was dramatically decreased.

Bone remodeling is tightly regulated by a molecular triad composed of OPG/RANK/RANKL, which has been considered as a connection pathway between osteoblast and osteoclastogenesis [29]. RANKL is mainly synthesized by the osteoblastic lineage cells, and is essential for mediating bone resorption through mediating osteoclastogenesis and the activation of mature osteoclasts [29,30]. The Western blotting analysis suggested that ODN MT01 might promote the proliferation and activation of MG 63 cells via the increasing ratio of OPG expression level to RANKL. In the present study, the expression level of OPG was improved, while RANKL expression level was significantly decreased.

## 3. Experimental Section

### 3.1. Materials

ODNs were obtained from College of Molecular Biology in Jilin University (Table 1), and then dissolved in axenic phosphate buffered solution (PBS). According to the functional characteristics, these ODNs could be divided into three types: (1) immunostimulant, including BW001 [31], FC001, BW006 [32], FC002, YW001 [33], FC004 and YW002 [34]; (2) immunosuppressant, including SAT05f [35], MT01 [35] and FC003; (3) immunologic inertia, such as MS19 [34]. Among them, BW001, BW006, YW001, YW002, SAT05f, MT01 and MS19 were once reported in our previous research [31–35], and others were first employed in the present research.

Human osteoblast-like cell line MG 63 was obtained from American Type Culture Collection (ATCC, CRL-1427), 3-(4,5-dimethylthiazol-2-yl)-2,5-diphenyl tetrazolium bromide (MTT) and dimethyl sulphoxide (DMSO) were purchased from Sigma-Aldrich (St. Louis, MO, USA). Alkaline phosphatase (ALP) kit and micro-BCA assay kit were obtained from Jiancheng Biological Reagent Co. (Nanjing, China). Real time PCR kit was purchased from TaKaRa (Tokyo, Japan). The monoclonal antibodies of anti β-actin, Sp7 and OPG were purchased from Santa Cruz Biotech. Co., and those of anti runx-2, collagen-I and RANKL were purchased from Abcam Co. (USA).

### 3.2. Cell Culture

The human MG 63 cell line was maintained in Dulbecco’s modified eagle’s medium (DMEM, Sigma, ST. Louis, MO, USA) containing 10% heat-inactivated fetal calf serum, 100 units/mL penicillin and 100 mg/mL streptomycin at 37 °C in a 5% CO_2_ fully humidified incubator. For subculture, cells at 80–90% confluence were passaged at a ratio of 1:3 after treating with 0.25% trypsin. When the cells (between 3th to 5th passage) have grown to semi-confluence, they were treated with different ODNs at a concentration of 1.0 μg/mL for 24 h, 48 h, 72 h, and 96 h.

### 3.3. MTT Assay

All the MTT assays were carried out in 96-well plates. Briefly, wells with 5000 cells/well were filled with 100 μL DMEM and 10% fetal calf serum (FCS), and 11 different ODNs (Table 1) were added to each well at a final concentration of 1.0 μg/mL. Control groups received no ODNs. The plates were incubated from 24 h to 96 h at 37 °C. After a brief wash with medium, 10 μL MTT (5 mg/mL) was added to each well, followed by incubation for additional 4 h. Finally, the supernatant was removed, and cells were lysed with 150 μL DMSO. The absorbance at 495 nm of each well was measured using a Bio RAD 550 automatic plate reader. The values were the average of triplicate measurements.

### 3.4. Measurement of ALP Activity

After treating with ODNs (1.0 μg/mL) for 24 h, 72 h and 120 h, cells were collected and lysed for ALP activity measurement. The experiment was conducted using Alkaline Phosphatase Kit, according to the manufacturer’s instructions. The protein concentration of cell lysates was measured using micro-BCA assay kit, and ALP activity was normalized for total protein concentration. The values were the average of triplicate measurements.

### 3.5. Real-Time PCR

Total RNA was isolated from MG 63 cells using the TRIzol reagent according to the manufacturer’s instructions. The purity of total RNA was determined by the ratio at 260 nm and 280 nm absorbance. 1 μg of total RNA was subjected to reverse transcription using RT-PCR Array First Strand Kit (SABioscience Co., USA). Following reverse transcription, each sample was diluted so that cDNA corresponding to the produced from 10 ng of total RNA was used in subsequent real-time PCRs. The primers (Table 2) were designed using qPrimerDepot, a primer database for quantitative real-time PCR and subsequently checked for specificity using BLAST (www.ncbi.nlm.nih.gov/genome/srq/HsBlast.html). PCRs were performed using the ABI Steponeplus (ABI PRISM, USA), which allowed real-time monitoring of the increase in PCR product concentration after every cycle based on the fluorescence of the double-stranded DNA specific dye SYBR green. The number of cycles required to produce a detectable product above background was measured for each sample. These cycle numbers were then used to calculate fold differences in starting mRNA level for each sample using the following method. First, the cycle number difference for GAPDH, a housekeeping gene, was determined in the control sample and appropriate ODN-treated sample. The 2^−ΔΔ*CT*^ method [36] was used for calculating the relative expression levels. The values were the average of triplicate measurements.

### 3.6. Western Blotting

MG 63 cells were cultured in 10 cm double dish and treated with the ODN MT01 (1.0 μg/mL) for predetermined time. Then the cells were collected, washed twice with Tris-buffered saline, and centrifuged at 5000 × g for 5 min at 4 °C. Afterwards, the collected cells were suspended in lysis buffer (50 mM Tris, pH 7.6, 0.01% EDTA, 1% Triton X-100, 1 mM PMSF, and 1 μg/mL leupeptin) on ice for 30 min, and centrifuged at 12000 × g for 15 min at 4 °C. The protein concentration was measured using BCA Protein Assay Reagent kit. The samples (40 μg protein) were separated by 12% SDS-PAGE and transferred to polyvinylidene difluoride (PVDF) membranes. The membranes were blocked with 5% skim milk in Tris-buffered saline with 0.1% Tween (TBST) for 1 h at room temperature, and followed by incubation with primary antibody at 4 °C overnight. Then the membranes were incubated with secondary antibody at 20 °C for 2 h, and detected using ECL chemiluminescent system. Loading differences were normalized using a monoclonal β-actin antibody.

### 3.7. Statistical Analysis

All experiments were performed on at least three individuals with each assay. The results were presented as mean ± SD. Using the Student’s t-test to analyze the data concerning the differences of gene expression. One-way ANOVA was applied to analyze the data concerning the cell proliferation and ALP activity. (A probability level of 5%, *P* < 0.05 was considered statistically significant).

## 4. Conclusions

In this paper, an ODN with no CpG, ODN MT01, was found to have a significant effect in facilitating osteogenic proliferation and activation, and this was probably due to the increasing ratio of OPG expression level to RANKL. This study provided a direct evidence for the notion that single strand ODN could regulate the balance of bone formation and resorption, and therefore has great potential in the rebuilding of alveolar bone.

## Figures and Tables

**Table 1.** ODNs (11 different sequences) were used for co-culture with MG 63 cells to screen the ones that could improve the proliferation of cells.

**Number**	**Name**	**Sequence**
1	SAT05f	5′-CCTCCTCCTCCTCCTCCTCCTCCT-3′
2	MS19	5′-AAAGAAAGAAAGAAAGAAAGAAAG-3′
3	BW001	5′-TCGTCGGGTGCGACGTCGCAGGGGGG-3′
4	FC001	5′-TCGGGGACGATCGTCGGGGAC-3′
5	FC002	5′-TCGTCGACGTCGTCGTTCTC-3′
6	BW006	5′-TCGACGTTCGTCGTTCGTCGTTC-3′
7	YW002	5′-TCGCGAACGTTCGCCGCGTTCGAACGCGG-3′
8	FC004	5′-TCGCGTTCGATCGCGATCGACGGTA-3′
9	MT01	5′-ACCCCCTCTACCCCCTCTACCCCCTCT-3′
10	YW001	5′-TCGCGACGTTCGCCCGACGTTCGGTA-3′
11	FC003	5′-TCTCTCTCTCTCTCTCTCTCTCTC-3′

**Table 2.** The primers used for qRT-PCR of various genes.

**Gene Name**	**Oligonucleotide UP (5′-3′)**	**Oligonucleotide DW (5′-3′)**
GAPDH	ATG GGG AAG GTG AAG GTC	TAA AAG CAG CCC TGG TGA CC
SP7	CAC AGC TCT TCT GAC TGT CTG CTG	GTG AAA TGC CTG CAT GGA T
RUNX-2	GAG ATC ATC GCC GAC CAC	TAC CTC TCC GAG GGC TAC C
COLLAGEN-I	AGG GCC AAG ACG AAG ACA TC	AGA TCA CGT CAT CGC ACA ACA
